# Public Medical Instruction

**Published:** 1875-04-15

**Authors:** 


### PUBLIC MEDICAL INSTRUCTION.

THE Medical Society of the County of Kings, N. Y., has appointed a standing committee, called the "Committee on Public Instruction." The chief duty enjoined on the committee is set forth in the following paragraph, quoted from the *Medical Record:*

"It is the purpose of the Committee of Public Instruction to communicate useful information on medical subjects to the public through the medium of the press. To this end, it shall obtain, prepare or select suitable papers, which it shall send for publication to such newspapers or periodicals (not medical) as it may select. The writer's name or other means of identification shall not accompany any original (specially-prepared) paper, nor shall the name of the author of any selected article be published when such author is a member of this or any neighboring society."

So far as relates to hygiene and sanitary regulations, such a committee, performing its work judiciously, would be of great benefit to the public in all our cities.

